# Biochemical Characterization of Kat1: a Domesticated *hAT*-Transposase that Induces DNA Hairpin Formation and *MAT*-Switching

**DOI:** 10.1038/srep21671

**Published:** 2016-02-23

**Authors:** Kishore K. Chiruvella, Naghmeh Rajaei, Venkateswara Rao Jonna, Anders Hofer, Stefan U. Åström

**Affiliations:** 1 Department of Molecular Biosciences, the Wenner-Gren Institute, Stockholm University, SE-10691 Stockholm, Sweden; 2 Department of Medical Biochemistry and Biophysics, Umeå University, SE-901 87 Umeå, Sweden

## Abstract

*Kluyveromyces lactis hAT*-transposase 1 (Kat1) generates hairpin-capped DNA double strand breaks leading to *MAT*-switching (*MAT***a** to *MAT*α). Using purified Kat1, we demonstrate the importance of terminal inverted repeats and subterminal repeats for its endonuclease activity. Kat1 promoted joining of the transposon end into a target DNA molecule *in vitro*, a biochemical feature that ties Kat1 to transposases. Gas-phase Electrophoretic Mobility Macromolecule analysis revealed that Kat1 can form hexamers when complexed with DNA. Kat1 point mutants were generated in conserved positions to explore structure-function relationships. Mutants of predicted catalytic residues abolished both DNA cleavage and strand-transfer. Interestingly, W576A predicted to be impaired for hairpin formation, was active for DNA cleavage and supported wild type levels of mating-type switching. In contrast, the conserved CXXH motif was critical for hairpin formation because Kat1 C402A/H405A completely blocked hairpinning and switching, but still generated nicks in the DNA. Mutations in the BED zinc-finger domain (C130A/C133A) resulted in an unspecific nuclease activity, presumably due to nonspecific DNA interaction. Kat1 mutants that were defective for cleavage *in vitro* were also defective for mating-type switching. Collectively, this study reveals Kat1 sharing extensive biochemical similarities with cut and paste transposons despite being domesticated and evolutionary diverged from active transposons.

Transposable elements (TEs) are mobile genetic elements colonizing the genomes of all studied organisms contributing substantially to evolution through their ability to move within genomes^1^. Their proliferation in host genomes can be remarkable, *i.e.* the Norwegian spruce genome consisting of >90% TEs and remnants^2^. TEs affect host genomes in multiple ways, perhaps most typically by inactivating or altering the regulation of host genes. However, these elements can also contribute to the adaptive evolution of host genomes^3^. Transposon domestication (also called exaptation) means that TEs acquire functions beneficial for the host. Examples include the donation of regulatory sequences thereby re-wiring regulation of host genes or that TE-encoding genes become utilized as transcriptional regulators or DNA recombinases^4^,^5^. There are two types of transposons, the retrotransposons that transpose through an RNA intermediate and the DNA transposons that use mostly a “cut and paste” mechanism for transposition^6^. Many DNA transposons contain a single gene encoding a protein called transposase. Transposases recognize the terminal inverted repeats (TIRs) of TEs and catalyze excision from their original position and integration into a new target site^7^. A catalytic triad consisting of the amino acids Asp, Asp, Glu (the last amino acid sometimes being Asp) defines the so-called DDE(D)-transposases, which are found in all kingdoms of life^8^. Retroviral integrases and DDE(D)-transposases share structural similarity, suggesting a common evolutionary origin^9^. Although this RNase H-like protein fold is conserved among DDE(D)-transposases and integrases, diverse members of these protein families also contain other domains that are unique to each^10^.

Hobo from Drosophila^11^, Ac from maize^12^ and Tam3 from the snap dragon^13^ are the founding members of the *hAT*-family of DNA transposons, which seems to be restricted to eukaryotes. This family has several distinguishing features, including 8-bp target site duplications and a carboxyl terminal ~50 amino acid domain called the *hAT*-domain (IPR008906)^14^. The *hAT*-domain was shown to be required for dimerization of the Activator transposase^15^. Recent structural studies of the Hermes transposase showed that the *hAT*-domain in fact did not represent an independent folding unit, but was important for knitting together different parts of the protein^16^. So far only a few *hAT*-transposases have been studied in detail, most notably Hermes from *Musca domestica*^16^,^17^,^18^. It was shown that Hermes excise through the generation of DNA double stranded breaks (DSBs) that are hairpin-capped on the flanking DNA^18^. This observation ties Hermes to the RAG1/2 recombinases that are essential for generating the vertebrate antigen receptors^19^. However, RAG1/2 are more closely related to *transib* elements than they are to *hAT*-transposases^20^. A recent crystal structure of Hermes bound to its TIRs revealed an octameric structure, consisting of a tetramer of dimers. It was suggested that the octameric nature of the Hermes:DNA assembly promoted end recognition and target capture by supplying multiple DNA-binding surfaces^17^.

Kat1 induces two hairpin-capped DSBs in the *mating type* **a** (*MAT***a**) locus in *Kluyveromyces lactis*^21^. These DSBs promote a gene conversion using the cryptic *hidden MAT left alpha* (*HML*α) mating type locus as donor, resulting in a switch of mating type from *MAT***a** to *MAT*α. Kat1 expression is induced transcriptionally by nutrient limitation, mediated by a nutrient-regulated transcription factor called Mts1. The *KAT1* gene contains a −1 frameshift that is bypassed by a programmed translational frameshifting event, which limits Kat1 expression. Hence, Kat1 is highly regulated and induces sexual differentiation in this budding yeast^21^.

Kat1 displays functional similarities with *hAT*-transposases, most notably generation of hairpin-capped DSBs. However, the amino acid similarity between Kat1 and *hAT*-transposases is limited. In addition, the sequences recognized by Kat1 remain elusive and the biochemical features of Kat1 have not been characterized. Here, using purified Kat1 transposase, we have studied the mechanism of DNA cleavage and have identified amino acids crucial for the catalytic steps. We found that several amino acids conserved among *hAT*-transposases were essential for Kat1 activity both *in vitro* and *in vivo*. However, the conserved Trp576 was not essential for activity, which was surprising given that the corresponding tryptophan in Hermes was important for the generation of hairpins. Even if Kat1 is domesticated and has lost its ability to transpose *in vivo*, we found that Kat1 performed strand transfer *in vitro*. Further, gas-phase electrophoretic macromolecule analysis (GEMMA) suggests that Kat1 can form a hexamer.

## Results

### Transposon end recognition by Kat1 transposase

**Figure 1:**
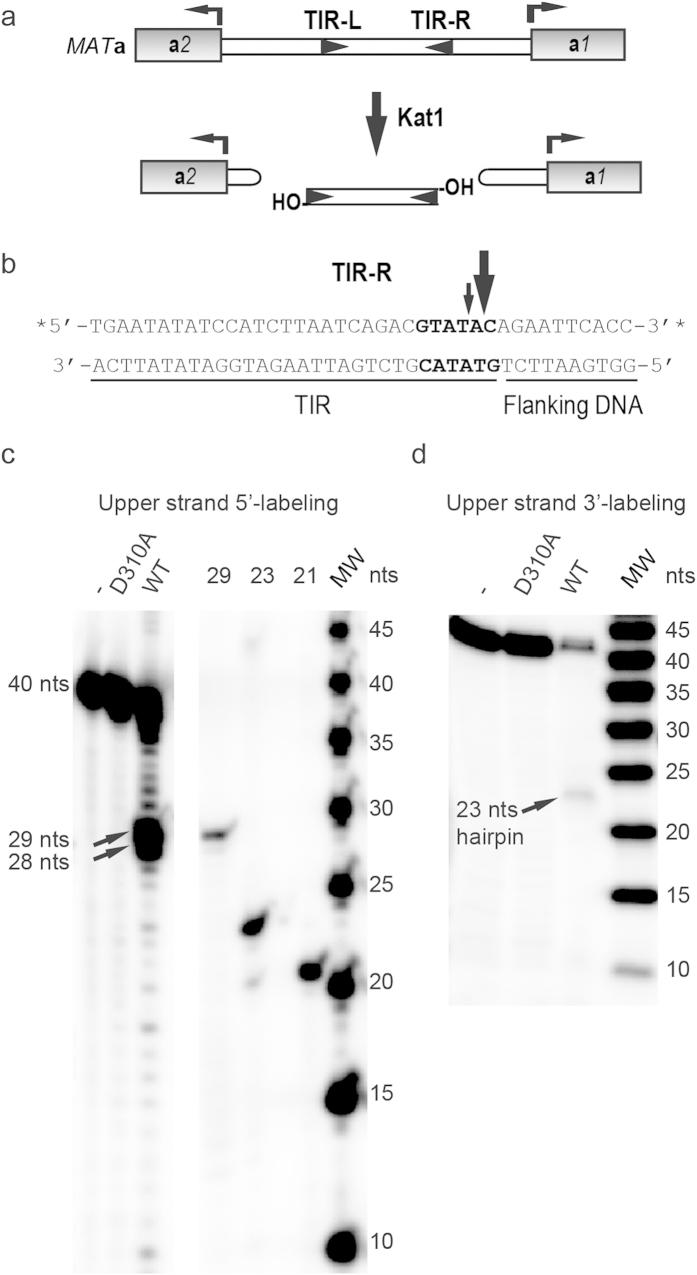
Kat1 cleaves TIR-R close to the transposon end. (**a**) Schematic drawing of the *MAT***a***1***-a***2* intergenic region displaying the **a***1* and **a***2* genes as grey boxes and the TIRs as arrowheads. Kat1 cleaves the TIRs, generating hairpin-capped DSBs on the flanking DNA and free 3′-OHs on the transposon ends. (**b**) The sequence of the 40-bp duplex representing TIR-R with 30-bp from the transposon end and 10-bp from the flanking DNA. The GTATAC sequence at the beginning of TIR-R is indicated in bold and major incisions on the upper strand represented by arrows. The asterisks (*) indicate the positions of ^32^P-label. (**c,d**) DNA cleavage assay with 5′end labeled (**c**), and 3′end labeled (**d**), DNA substrates using WT Kat1, Kat1D310A and no protein (−). Each reaction, containing ~8 nM of substrate and ~200 nM of Kat1, was incubated for 3 hrs at 30 °C, deproteinized and analyzed by 15% denaturing PAGE. The radiogram depicts the 40-nt substrate, the major products formed and size markers. A representative image from at least two independent experiments is shown.

The frameshift of the endogenous *KAT1* gene was corrected, generating a *KAT1+G* allele as described before^21^. A Glutathione S-transferase (GST)-tagged Kat1+G (from now on called GST-Kat1) was expressed in bacteria (*Escherichia coli*), followed by purification using glutathione Sepharose 4B. We have previously shown that Kat1 requires two hexameric sites (GTATAC) in the *MAT***a***1***-a***2* intergenic region (Fig. 1a) for generating DSBs^21^. The GTATAC sequences are the beginning of two imperfect inverted repeats, called TIR-R and TIR-L that are separated by 385-bp of DNA defining a nonautonomous TE (Fig. 1a). In addition, the DSBs generated were hairpin-capped on the flanking host DNA, but the DNA products on the transposon side were readily denatured into single stranded DNA of the expected size^21^.

To pinpoint the cleavage site, GST-Kat1 was mixed with a 40-bp duplex representing TIR-R. The 40-bp duplex included 30-bp from the transposon end and 10-bp from the flanking DNA. The products from the reaction were separated on a 15% denaturing PAGE, allowing single nucleotide resolution. To visualize both ends of the DSB, the upper strand (as drawn in Fig. 1b) was labeled separately on the 5′ and 3′ ends, followed by annealing to the unlabeled lower strand. Oligonucleotides (29, 23 and 21-nts) were used as size markers together with a 5-bp ladder. Kat1 generated a major product of 29-nts and a minor product of 28-nts on the upper strand labeled on the 5′ end (Fig. 1c). This showed that Kat1 cleaved the upper strand primarily at GTATA*C and to less extent at GTAT*AC. In the reaction using 3′ end-labeling of the upper strand a product of 23-nts was observed (Fig. 1d). We envision that this product is generated by nicking the lower strand, followed by the 3′-hydroxyl attacking the upper strand generating a DNA hairpin. The length of this hairpin was expected to be 22-nts plus 1-nt as a result of the labeling with terminal deoxynucleotidyl transferase, hence confirming the position of cleavage. The products formed from the lower strand are hairpin-capped (Supplementary Fig. S2). Importantly, we observed no cleavage products when the substrate was incubated with catalytically inactive Kat1D310A, showing that the observed cleavage products were produced by Kat1 and not by contaminating bacterial nucleases. These data showed that Kat1 cuts TIR-R in the GTATAC site *in vitro*.

**Figure 2:**
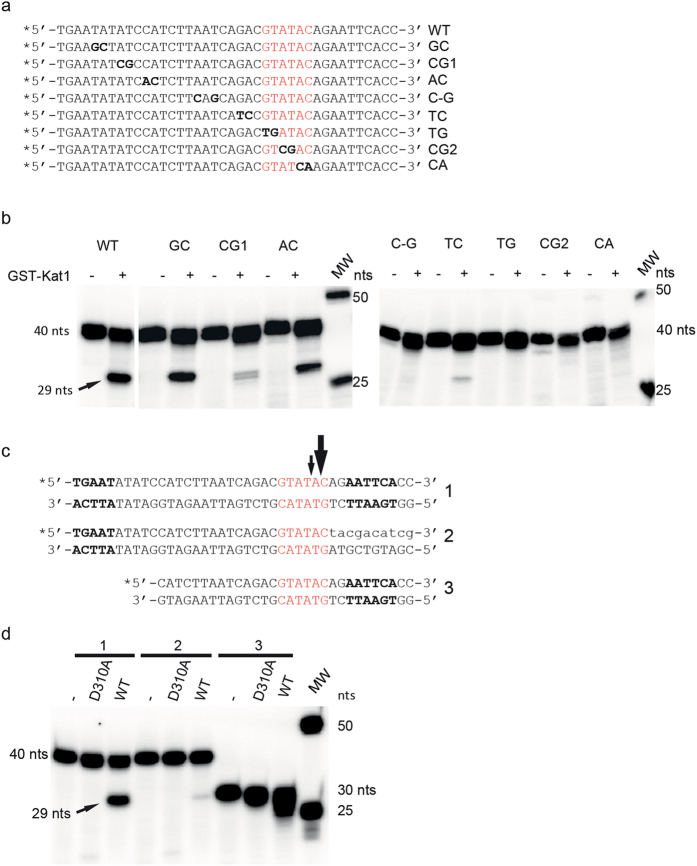
The Kat1 recognition site in TIR-R extends beyond the GTATAC sequence. (**a**) DNA sequences showing the upper labeled strand of 40-bp duplexes containing mutations in TIR-R. Mutations are indicated in bold letters and designated WT, GC etc. The GTATAC sequence at the beginning of TIR-R is indicated in red. (**b**) The indicated mutant DNA substrates were incubated with Kat1 (+) or without protein (−) using the conditions described in Fig. 1, except that the gel was thicker. The 40-nt substrates, the 29-nt products and DNA ladder (MW) is shown. (**c,d**) The Kat1 endonuclease activity of TIR-R requires a flanking DNA sequence. (**c**) Representation of DNA duplexes used as substrates, the WT 40-bp duplex (1), a 40-bp duplex with 10-bp scrambled on the flanking DNA end (2) and a 30-bp duplex (3). Arrows indicate incisions on the top strand, asterisks positions of ^32^P-label (upper strand was labeled) and bold letters indicate subterminal repeats. (**d**) Nuclease assay using WT Kat1, Kat1D310A and no protein (−). Reaction conditions were as described in Fig. 1. The radiogram depicts the 40-nt and 30-nt substrates, the 29-nt product and size markers. Note that both the 30-bp substrate and 40-bp substrate with a scrambled host end lack subterminal repeats TGAAT and AATTCA, respectively.

Notably, Kat1 did not display activity towards a short 40-bp duplex representing TIR-L, preventing us from probing the exact cleavage site. However, we could demonstrate cleavage of TIR-L using a longer substrate (212-bp) (Supplementary Fig. S3), and the length of the products showed that Kat1 cleaved also TIR-L close to the beginning of the TIR. TIR-L and TIR-R have several identical residues in addition to the GTATAC sequences. Moreover, aligning the *MAT***a***1-MAT***a***2* intergenic region from *K. lactis* with the corresponding regions in *K. marxianus* and *K. dobzhanskii* (evolutionary closely related species) showed that several of the bases in the TIRs were conserved (Supplementary Fig. S4). To extend our analysis and ask whether other bases were important for Kat1 cleavage, a series of duplexes with mutations in TIR-R were generated (Fig. 2a). The mutant duplexes contained point mutations from the beginning of TIR-R, extending 26-bp into the transposon. Strikingly, base substitutions in the GTATAC sequence abolished Kat1 cleavage (Fig. 2b, right panel). Other mutations in the TIR also decreased Kat1 cleavage to various extents (Fig. 2b, left panel). These data demonstrate the importance of an extended sequence in TIR-R for promoting Kat1 cleavage, probably explaining why Kat1 cleaves only the *MAT***a** locus and not elsewhere in the genome.

### Potential role of subterminal repeats for Kat1 activity

To address why the 40-bp TIR-R duplex was a better substrate for Kat1 than the 40-bp TIR-L duplex, two additional TIR-R substrates were generated. One substrate was a 40-bp duplex, in which the 10-bp flanking TIR-R (the “host” DNA) was scrambled (Fig. 2c, substrate 2). The other substrate was a TIR-R duplex only 30-bp long, removing an additional 10-bp from the transposon end (substrate 3). Neither of the substrates were cut by Kat1. That the 30-bp duplex was a poor substrate for Kat1 was not surprising, since the point mutant analysis showed that bases that were deleted were important for Kat1 activity. The results with the substrate containing scrambled flanking DNA indicated that there were bases on the “host side” of TIR-R that were important for cleavage (Fig. 2d). The multiple sequence alignment using the *MAT***a***1*-**a***2* intergenic region revealed that apart from conservation of the TIRs there was a hexameric sequence (AATTCA or its complement TGAATT) that was repeated six times between TIR-R and TIR-L (Supplementary Fig. S4). These subterminal repeats were also conserved in all three *Kluyveromyces* species aligned, indicating that they might be important. In the *K. lactis* sequence, one such subterminal repeat was present in the “host” DNA that was scrambled in the mutant above. Since this mutant was a poor substrate for Kat1, despite having an intact 30-bp TIR-R (Fig. 2d), we speculate that these subterminal repeats might enhance the Kat1 endonuclease activity. Earlier studies of other *hAT*-transposases have reported that transposon ends contain several subterminal repeats and that such repeats modify transposase activity^17^,^22^,^23^.

### Kat1 promotes strand-transfer *in vitro*

**Figure 3:**
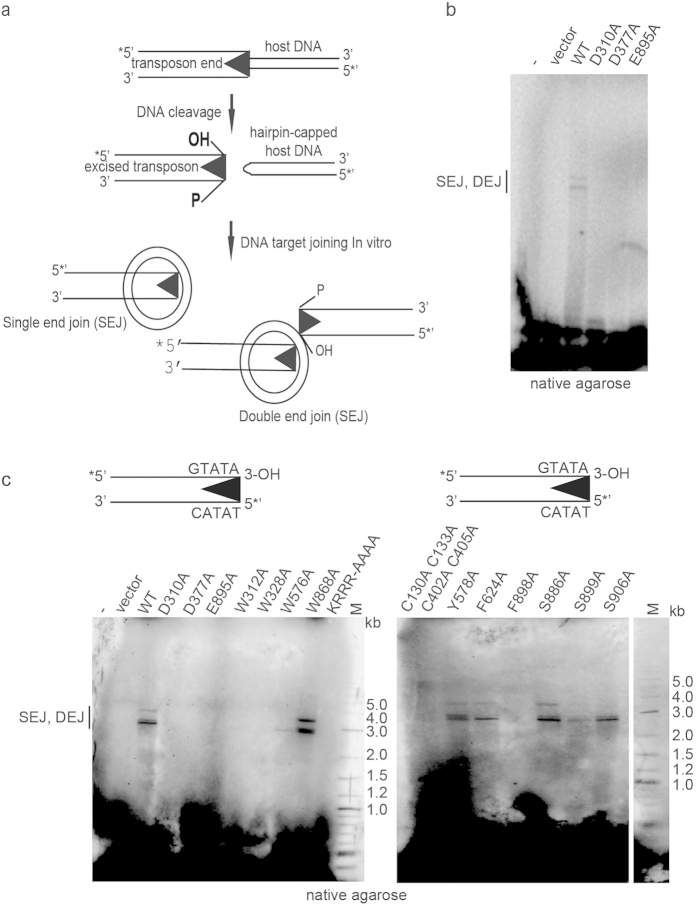
Kat1 catalyzes strand-transfer. (**a**) Schematic diagram of Kat1 cleavage. A DSB is formed between the flanking DNA and the transposon end. The products are a transposon end containing a free 3′-OH and hairpin-capped flanking DNA. Joining of a single 3´-OH end to target DNA leads to formation of a single end join (SEJ) product, whereas joining of two transposon ends to opposite strands of the target results in a double end join (DEJ) product. (**b**) Strand-transfer assay using WT Kat1 and DDE mutants as indicated, a ^32^P-labeled PCR-product corresponding to TIR-R (197-bp, ~6 nM) and a circular target plasmid (pUC19). (**c**) Kat1 strand-transfer using pre-cleaved substrate. Schematic representation of 122-bp PCR amplified (using primers KKC55 and KKC52) DNA substrate with free 3´-OH transposon ends. The pre-cleaved transposon end (~10 nM) was incubated with pUC19, WT Kat1 and a panel of Kat1 mutant proteins. In all cases, samples were incubated with transposase for 3 hrs at 30 °C, deproteinized and separated on 1.3% native agarose gel.

Retroviral integrases and transposases catalyze a strand-transfer reaction, in which the TE or retrovirus is inserted into the host genome. The strand-transfer activity of a transposase can be tested *in vitro* by mixing a transposase with the TE and a target DNA molecule. We used pUC19 as target and included Kat1 and a 197-bp duplex corresponding to TIR-R with 48-bp from the transposon and 149-bp flanking DNA. In this assay, first the transposase cleaves the transposon end, generating a free 3′ hydroxyl (3′-OH). Joining of one 3′-OH to the target generates a nicked plasmid or single end-joining (SEJ) product. If two 3′-OHs join the target at complementary positions on opposite strands, the plasmid is linearized generating a double end-joining (DEJ) product (Fig. 3a). Kat1 displayed target joining, generating both SEJ- and DEJ-products on a native agarose gel. As expected, Kat1 with mutations in the catalytic DDE-motif (D310A, D377A and E895A) did not show detectable target joining (Fig. 3b) even when using a pre-cleaved transposon end substrate, in which the terminal 3-OH was already exposed (Fig. 3c, left panel). Thus, Kat1 retains the strand-transfer activity characteristic of transposases.

### Kat1-DNA interactions

**Figure 4:**
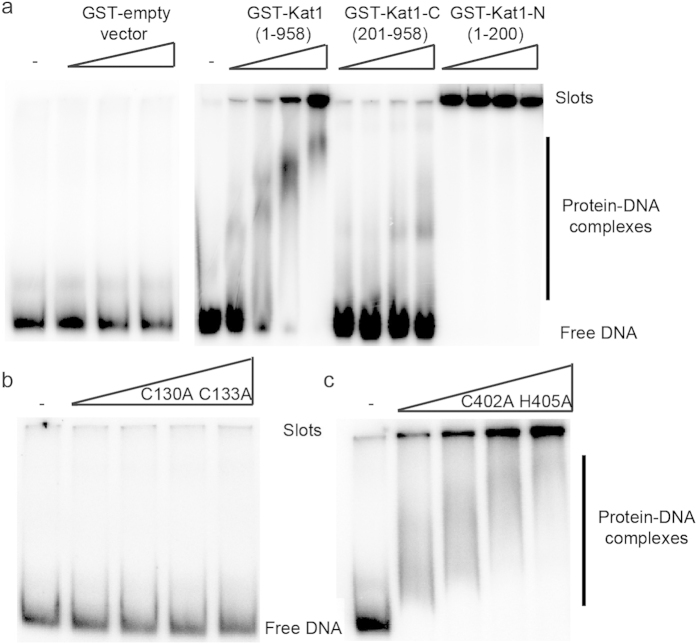
The Kat1 BED-domain is important for DNA binding *in vitro*. Electrophoretic mobility shift assay (EMSA) using a 577-bp DNA probe including both TIR-R and TIR-L and increasing amounts of (**a**) GST-empty vector, GST-Kat1, GST-Kat1-C and GST-Kat1-N. Free probe, protein-DNA complexes and slots are indicated. (**b,c**) as in (**a**), but using increasing amounts of GST-Kat1C130A/C133A and GST-Kat1C402A/H405A. In each reaction ~1.4 nM of substrate was incubated with ~5, 10, 20, and 40 nM of Kat1 for 1 h at 4 °C. Reaction products were analyzed by 4% native PAGE.

To characterize the DNA-binding of Kat1, electrophoretic mobility shift assays (EMSAs) were performed. Full-length GST-Kat1 bound to a 577-bp probe that included both TIR-L and TIR-R. However, there were no specific band shifts, but rather a smear of slower migrating protein-DNA complexes that with increased concentration of GST-Kat1 shifted all of the probe into the well (Fig. 4a). This indicated formation of multimeric complexes, containing more than one GST-Kat1 molecule. Other experiments showed that the DNA binding was unspecific, demonstrating that cold non-specific Drosophila DNA competed for the mobility shift with equal efficiency as the cold *MAT***a** probe (Supplementary Fig. 5). Transposases often display unspecific DNA binding activity in agreement with their ability to interact with target DNA to insert the TE at a new locus. We predicted that the DNA-binding domain of Kat1 would reside in its N-terminus based on comparisons between Kat1 and other previously characterized *hAT*-transposases. To address this possibility, the first 200 amino acids of Kat1 (Kat1-N) were expressed separately as a fusion to GST. As control, we expressed the central and C-terminal domains of Kat1 (amino acids 201–958, Kat1-C) as a separate fusion protein (Supplementary Fig. S8). Consistent with the idea that Kat1 interacted with DNA via the N-terminus, Kat1-N bound to the probe, whereas Kat1-C did not (Fig. 4a). Interestingly, the binding was much stronger with the N-terminal domain alone than with the full length protein, indicated by the probe shifting completely (Fig. 4a).

The N-terminus of Kat1 contains a so called BED-finger (PF02892)^24^, also found in other transposases, where cysteines 130 and 133 were predicted to have a key role in Zn^2+^-ion coordination. BED-fingers have been predicted or shown to be essential for DNA binding in other transposases^17^,^25^,^26^. Moreover, an additional motif potentially capable of cysteine coordination is found in the middle of the protein (C402 and H405). This prompted us to examine DNA binding of Kat1 C130A/133A and C402A/H405A mutants in the EMSA. The full-length GST-Kat1 C130A/133A mutant lost detectable DNA binding (Fig. 4b), whereas the C402A/H405A mutant bound DNA normally (Fig. 4c). In conclusion, Kat1 bound DNA non-specifically and the DNA interaction was dependent on a Zn^2+^-finger motif in its N-terminus.

### Kat1 forms a hexamer

**Figure 5:**
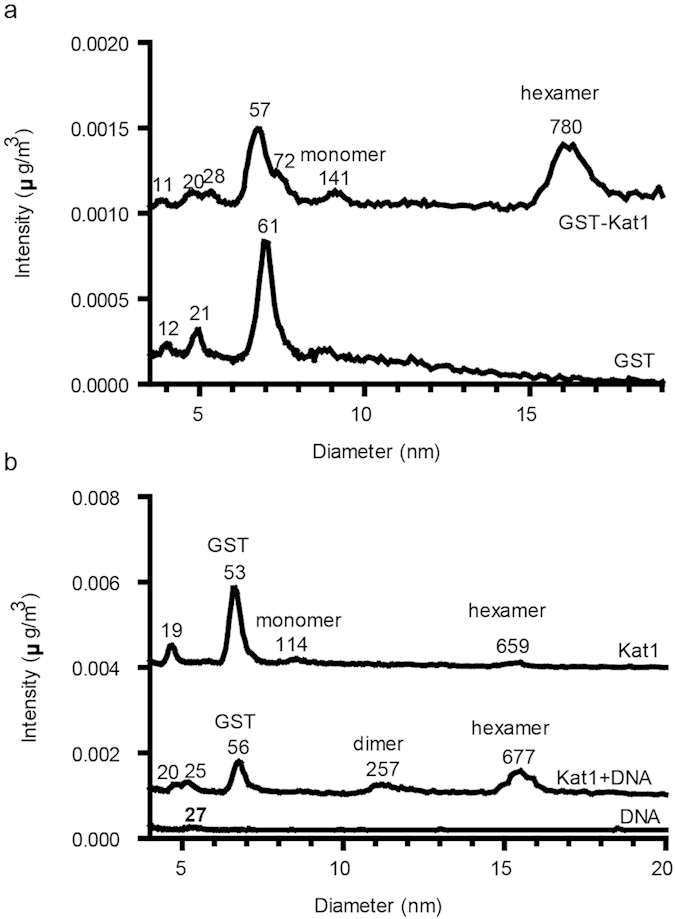
Formation of oligomeric Kat1 assemblies. (**a**) GEMMA analysis of GST-Kat1 (top, calculated MW 136 kDa) and GST (bottom, calculated MW 26.9 kDa). (**b**) GEMMA analysis of 0.085 mg/ml Kat1 alone (top trace, expected monomeric MW 109 kDa), Kat1 together with a 40-bp duplex TIR-R DNA (middle trace) or 0.18 μM of the duplex DNA alone (bottom trace). In the experiment with Kat1 and DNA (middle trace), they were incubated together for 2h at a concentration of 0.43 mg/ml Kat1 (4.0 μM) and 1.4 μM DNA. Next, the mixture was desalted to 100 mM ammonium acetate by gel filtration, diluted twice in water and analyzed by GEMMA (the final protein concentration was approximately 0.05 mg/ml). The processed data in (**a**,**b**) displays a mass‐based concentration scale with the baselines of the upper traces shifted in order to fit many experiments in each graph. Molecular masses are indicated on top of the peaks and peaks representing GST and different oligomeric structures of Kat1 are indicated.

Transposases invariably oligomerize with at least one active site positioned at each transposon end. We predicted that Kat1 might oligomerize as other *hAT*-family members such as TOL2TP, Hermes and Activator form tetramers, octamers and dimers, respectively^15^,^17^,^27^. In addition, the EMSA results using Kat1 suggested that higher-order protein-DNA complexes were formed. We analyzed the oligomeric status of DNA-free Kat1 transposase by GEMMA. This technique is used for determining the molecular weight of protein complexes^28^,^29^,^30^. GST-Kat1 displayed several peaks (Fig. 5a), particularly interesting was a peak of 780 kDa, which supported the notion that the protein can oligomerize. The mass of GST-Kat1 is ~136 kDa, so the peak at 780 kDa was consistent with Kat1 existing as a hexamer. Since the GST‐Kat1 polypeptide is considerably larger than 100 kDa, most other peaks should be impurities. Analysis of GST alone showed a major peak of 61 kDa supporting that GST forms dimers. It also shows two minor peaks of 12 and 21 kDa that probably are impurities (Fig. 5a).

To rule out the possibility that the GST-tag affected the results, the GST-tag was cleaved from the GST-Kat1 fusion protein using thrombin protease (Supplementary Fig. S6). GEMMA analysis of non‐tagged Kat1 (109 kDa) showed similar peaks as the GST‐tagged Kat1. However, the intensity of monomeric and hexameric peaks were decreased dramatically (Fig. 5b, top trace). As expected, the molecular masses of monomeric and hexameric Kat1 were decreased due to removal of the GST-moiety. The strong peak at 53 kDa was probably the cleaved off GST protein. When Kat1 was mixed with the 40-bp duplex of TIR-R (molar ratio 2.8 of Kat1 polypeptide vs. DNA), the Kat1 peaks shifted slightly to higher mass and became more intense. We could also observe a new peak of 257 kDa that might represent a Kat1 dimer (Fig. 5b, middle trace). The reason why Kat1 itself gives a very weak signal in GEMMA might be due to that it is absorbed by the capillary and is not sprayed out in the gas‐phase efficiently. This problem seems to be partly relieved by the binding of DNA to the protein or by having the GST-tag attached to the protein. In conclusion, the GEMMA data shows that Kat1 can oligomerize and is most likely a hexamer.

### Kat1 amino acids crucial for DNA cleavage activity

**Figure 6:**
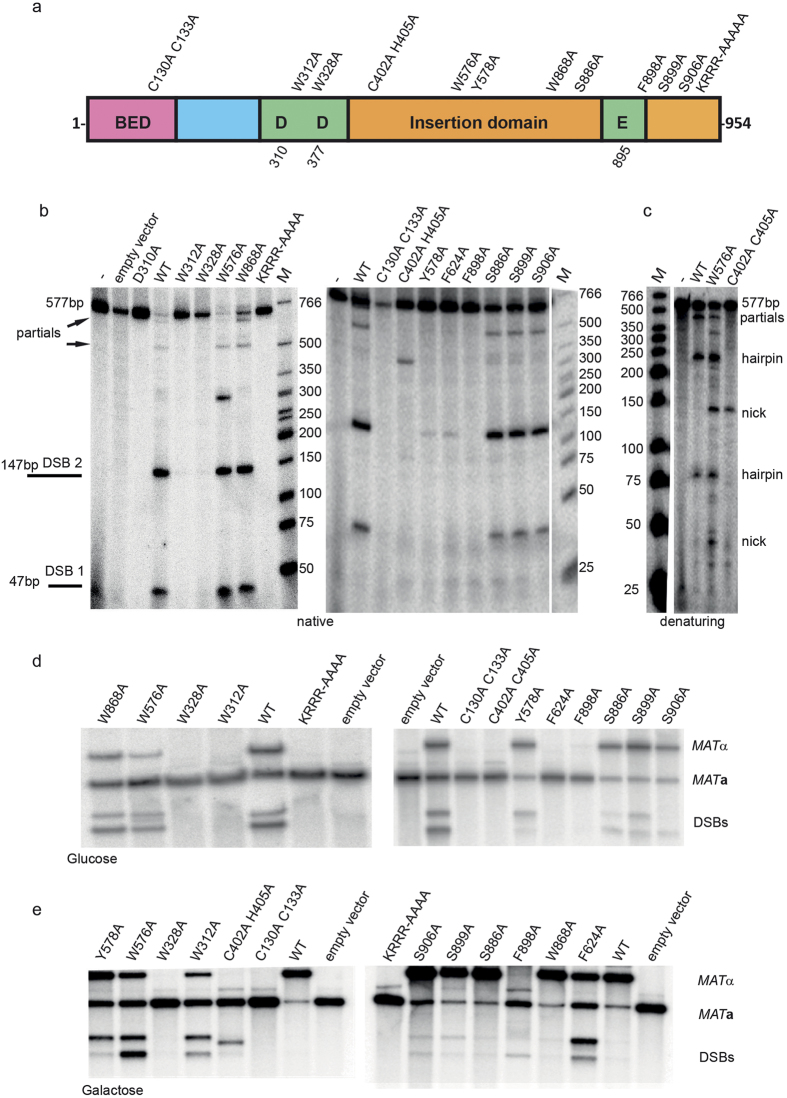
Importance of conserved amino acids for Kat1 function *in vitro* and *in vivo*. (**a**) Drawing of the functional domains of Kat1. Point mutations in Kat1 analyzed are depicted above the boxes and the positions of the catalytic DDE-residues indicated below. (**b**) *In vitro* cleavage assay (as in Fig. 1) with a panel of Kat1 mutations using GST-Kat1 mixed with a 577-bp substrate including both TIR-R and TIR-L, followed by separation on 6% PAGE using native conditions. The position and expected sizes of products, including partials are indicated on the left. Molecular weight designated M is also shown. (**c**) *In vitro* cleavage assay using C402A/H405A and W576A Kat1 mutations as in (**b**), but separated using denaturing conditions. The substrate and products (nicks and hairpins) are indicated. Both DNA strands were labeled in their 5′- ends and incubated with transposase for 3 hrs at 30 °C. (**d,e**) DNA-blot analysis of BamHI-digested genomic DNA from strain SAY1597 (*kat1*∆) carrying plasmids expressing the indicated Kat1 mutants using the galactose-inducible *GAL1* promoter. The blot was hybridized with a *MAT*-specific probe. Supplementary Fig. S9 shows a schematic diagram of the *MAT***a** and *MAT*α loci. The *MAT*α, *MAT***a**, and DSB-bands are indicated on the right. In (**d**) the strains were grown in glucose (low Kat1 expression) whereas in (**e**), galactose was used as carbon source (high Kat1 expression).

Evidence from structural, biochemical and mutational analyses of transposases have provided a framework to pinpoint the important amino acid residues crucial for nicking, DNA hairpin formation and strand transfer^16^,^17^,^18^,^31^,^32^,^33^. As a prelude for a mutational analysis of Kat1, we aligned Kat1 to Hermes and the fungal *hAT*-transposases Tfo1, Drifter and Restless (Supplementary Fig. S7)^15^,^17^,^18^,^34^,^35^,^36^,^37^. This analysis revealed only a handful of conserved residues, consistent with previous analyses of *hAT*-transposases^14^. We recognized an N-terminal BED-domain, the DDE-motif and a so-called insertion domain separating the aspartates in the DDE-motif from the glutamate residue (Fig. 6a).

Comparison to the Hermes crystal structure indicated that several of the conserved residues in Kat1 were predicted to be in direct contact with the TIRs or part of the active site. In an earlier study, we showed that mutations in the DDE-motif (D310A, D328A and E895A) of Kat1 completely abolished the endonuclease activity^21^. We generated a total of 13 mutants by site directed mutagenesis, expressed and partially purified them from bacteria and found that the steady-state levels of all mutants were comparable to that of WT Kat1 (Supplementary Fig. S8). Next we tested if these mutants could cleave their target sites using a 577-bp long substrate that included both TIR-R and TIR-L. Kat1 cleavage at TIR-L generates a ~47-bp hairpin-capped fragment, whereas cleavage at TIR-R generates a ~147-bp hairpin. The analysis shown in Fig. 6b used native conditions and the hairpins thus migrate as ~47 and ~147-bp fragments, respectively. The cleavage by WT Kat1 was efficient and only small amounts of uncleaved and partially cleaved products (cleaved at only one of the sites) were observed.

The C130A/C133A mutant did not cleave the substrate DNA. This was consistent with the observation that this mutant had diminished DNA-binding (Fig. 4b). However, we observed an unspecific degradation of the 577-bp substrate in multiple experiments (Fig. 6b, right panel). We interpret this to mean that in these conditions with a high Kat1 C130A/C133A/substrate ratio, the enzyme still retains some of its unspecific DNA interaction, but has lost the specific interaction to the TIRs. This resulted in degradation (unspecific cleavage) of the substrate.

The charged residues K907, R909 and R911 in Kat1 corresponds to K585, R586 and R588 in Hermes, which are in contact with the TIRs in the Hermes crystal structure^17^. We generated a K907A/R908A/R909A/R911A quadruple mutant (called KRRR-AAAA), which was completely inactive in the cleavage assay (Fig. 6b, left panel). H268 in Hermes is part of a conserved CXXH-motif, conserved in several transposase superfamilies. In Hermes, H268 was shown to be part of the active site^17^ and mutations in H268 resulted in a severe defect of the catalytic activity^18^. We generated a mutation in the Kat1 CXXH motif, C402A/H405A, which resulted in a severe defect in hairpin formation, consistent with the data from Hermes (Fig. 6b, right panel).

W312 and W576 of Kat1, correspond to the W182 and W319 in Hermes, respectively. W182 and W319 in Hermes are both close to the active site and are required for efficient hairpin formation^17^. A model from bacterial Tn5, which also forms hairpin-capped DSBs, although the hairpins are generated on the transposon side of the break, posits that aromatic amino acid residues can form stacking interactions with a flipped-out base on the opposite strand from where the initial nick is made^38^,^39^,^40^. This stacking interaction is suggested to facilitate the attack on the opposite strand, hence being important for DNA hairpin formation. In Rag1, aromatic amino acids are important for hairpinning, but such residues are probably not involved in a stacking interaction to a flipped-out base^41^. We investigated the importance of conserved aromatic residues in Kat1 including W312, W576, Y578, F624, W868 and F898 as well as the nonconserved W328. We generated single alanine substitution mutants for all of these residues. The *in vitro* cleavage assay showed that the W576A and W868A cleaved both TIR-R and TIR-L with an efficiency similar to WT Kat1 (Fig. 6b, right panel). The Y578A and F624A mutants cleaved the substrate with lower efficiency, whereas the W312A, W328A and F898A mutants displayed no detectable cleavage at all (Fig. 6b). Thus, several conserved aromatic residues in Kat1 are important for activity.

Two of the mutants tested, W576A and C402A/H405A displayed a product in the cleavage assay that was not observed using WT Kat1 (Fig. 6b). The size of this product was inconsistent with it being a partial digestion of the substrate and we hypothesized that it might be a nick of the transposon end. To test this idea, we used a denaturing gel to separate the reaction products (Fig. 6c). This analysis showed that the W576A and C402A/H405A mutants generated two single stranded fragments of ~47 and ~147-nts. The W576A mutant also generated the expected hairpins, but the C402A/H405A mutant exclusively generated nicked products. We conclude that the C402A/H405A mutant was critical for hairpin formation, but not for nicking, and speculate that the coupling between nicking and hairpinning was compromised in the W576A mutant.

Finally, the C-terminus of Kat1 contains several conserved serines. To examine the importance of these serines, we generated S886A, S899A and S906A mutants. All mutants retained catalytic activity similar to WT Kat1 (Fig. 6b, right panel).

### Strand-transfer activities largely mimicked nuclease activity

The 13 mutants were tested in the strand-transfer assay using a pre-cleaved transposon end substrate, in which the terminal 3-OH was exposed (Fig. 3c). The results obtained closely mimicked the results from the *in vitro* cleavage assay, *i.e.* Kat1 mutants that were severely or partially impaired for cleavage were also inactive for target joining. This is consistent with the notion that the same active site in Kat1 performs both the cleavage and strand-transfer reactions. There was one exception, however, the W576 mutant was capable of generating hairpins, but its strand-transfer activity was barely detectable (Fig. 3c).

### Kat1 amino acids critical for DSB formation *in vivo*

Expression of Kat1 results in mating-type switching from *MAT***a** to *MAT*α in *K. lactis*. Therefore, to learn about the *in vivo* consequences of mutations in *KAT1*, we measured the efficiency of mating-type switching using the 13 mutants described above. The point mutations were generated in an expression vector that used the galactose-inducible *GAL1* promoter to drive Kat1 expression and then introduced into a *kat1*Δ strain. Protein-blots of whole cell lysates showed that the Kat1 mutants were stably expressed displaying similar levels as WT Kat1 (Supplementary Fig. S9A). The *GAL1* promoter is leaky in *K. lactis* resulting in lower expression in glucose-grown cells and higher expression in galactose-grown cells. Comparing mating-type switching in galactose and glucose grown cells can thus tease out differences in the activity of Kat1 mutants.

**Figure 7:**
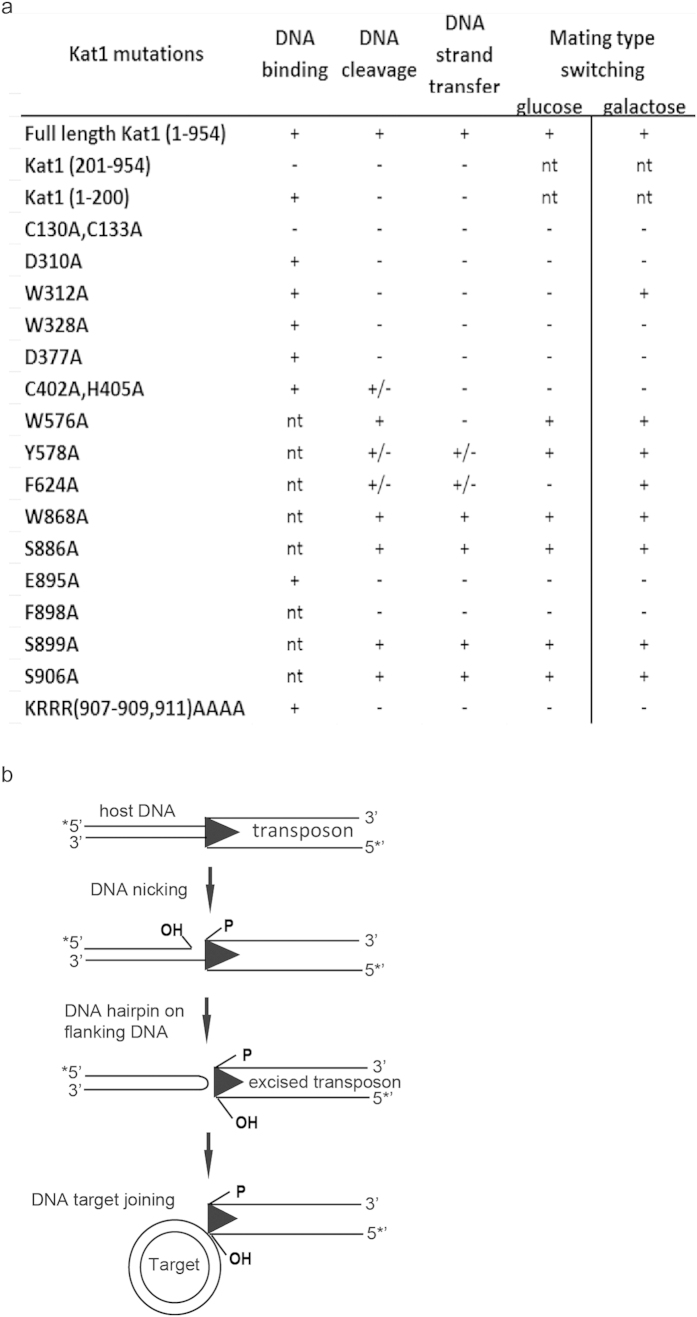
Kat1 displays extensive similarities to cut and paste DNA transposons. (**a**) Table summarizing the mutational analysis of Kat1, with respect to DNA binding, DNA cleavage, DNA strand transfer and mating-type switching in glucose and galactose *in vivo*. “+”, indicates active; “−”, not active; “+/−”, partially active; “nt”, not tested. (**b**) Mechanism of action of Kat1 transposase. *In vitro*, Kat1 is a cut and paste DNA transposase cleaving the transposon end by specific sequence recognition and strand-transfers the excised DNA into target DNA.

We performed DNA-blots using a *MAT*-specific probe (Supplementary Fig. S9B). The strain containing WT Kat1 grown in glucose displayed four hybridizing bands corresponding to the *MAT***a** and *MAT*α loci as well as switching intermediates corresponding to DSBs in either TIR-R or TIR-L (Fig. 6d and Supplementary Fig. S9B). The WT Kat1 strain grown in galactose had converted to *MAT*α almost completely and the DSB-intermediates were not detectable (Fig. 6e). The Kat1 mutants C130A/C133A, W328A, C402A/H405A, F898A and KRRR-AAAA displayed no *in vivo* activity. The serine mutants (S886A, S899A and S906A) and W868A displayed *in vivo* activity similar to WT Kat1. For the remaining mutants, the activity was compromised, but not absent. As shown in Fig. 6d, W312A and F624A had no detectable activity in glucose, but could induce mating-type switching when overexpressed (Fig. 6e). We conclude that the results obtained from the *in vivo* assay were in excellent agreement with the results from the *in vitro* assays, confirming the validity of the results. The activity of the Kat1 mutants in the different assays is summarized in Fig. 7a.

## Discussion

We characterized the Kat1 protein, mediating *MAT*-switching in the budding yeast *K. lactis*, and found that it possesses typical features of transposases. It generates sequence specific DSBs at TIRs that most likely are remnants of an ancient transposition event. It binds DNA nonspecifically and can transfer DNA strands from the transposon end into a target DNA molecule *in vitro*. By these criteria, Kat1 is a typical transposase performing “cut and paste” transposition (Fig. 7b). However, there is no evidence that Kat1 mediates transposition *in vivo*, rather it generates DSBs that are used as primers for a gene conversion leading to a switch of mating type. If Kat1 would mediate transposition, then it would be expected to leave remnants of transposition events in the *K. lactis* genome, but no such remnants are evident from extensive BLAST-analyses. Hence, even if Kat1 performs all the biochemical steps required for transposition *in vitro*, it is unlikely to do so *in vivo*. This is similar to other domesticated transposases such as the human SETMAR protein^42^.

The BED-domain was found to be important for DNA binding in mobility shift assays. However, it is unlikely that the C130A/C133A mutation completely abolished Kat1-DNA interaction (Fig. 4b), since the mutant protein displayed an unspecific degradation of the DNA substrate used in the *in vitro* cleavage reaction (Fig. 6b). This indicated that other domains than the BED-domain facilitate Kat1-DNA interaction, but that the BED-domain is important for a specific interaction with the DNA substrate. In contrast, earlier studies of Hermes showed that the BED-domain was not required for specific interaction to the target site, DNA cleavage or strand transfer^16^. Since different transposases recognize different DNA motifs, it is not surprising that DNA-binding domains of transposases evolve faster than the active sites. Nonspecific DNA binding has been documented previously for transposases, recombinases and integrases^43^,^44^.

GEMMA analyses indicate that Kat1 forms a hexamer in solution. Whether the proposed hexamer of Kat1 is in equilibrium with other quaternary forms must await further experiments. The active form of Hermes is probably an octamer since Hermes variants capable of forming only dimers were active for cleavage and strand transfer *in vitro*, but inactive for transposition *in vivo*^17^. In addition, oligomerization of several transposases/integrases, for example the Mos1 and MuA transposases^45^,^46^ and HIV-1 integrase^47^, have been linked to function. Multimeric states of transposases coupled with competition for binding sites to the transposon ends have also been suggested to control TE copy number^48^. Transposase-DNA complexes will almost certainly always have at least two active sites that promote cleavage and strand-transfer on the two ends of the element. However, the number of monomers in active transposase-DNA complexes varies between different TEs. The interface between dimers in Hermes, which mediates the formation of octamers, is not conserved in Kat1. Hence, the oligomerization of Kat1 most likely use a different monomer interface compared to Hermes.

Exploring DNA sequence requirements for Kat1 activity using mutated and truncated versions of TIR-R revealed that Kat1 cleavage requires an extended sequence for efficient cleavage. This observation probably explains why Kat1 cleaves only the *MAT***a** locus and not elsewhere in the genome. The conserved GTATAC sequence was essential since mutations in this sequence abolished cutting. We hypothesize the lower strand is nicked first (as drawn in Fig. 1b), and then the resulting 3′-OH attacks the upper strand, generating a hairpin. This occurs mostly 1-nt from the beginning of the TIR, generating a hairpin of 22-nts (Fig. 1d) and a transposon end of 29-nts (Fig. 1c). Sequence analysis revealed that the sequence TGAATT was present 6 times between TIR-L and TIR-R. Considering 5/6 matches, it is present 12 times, with several of these subterminal repeats being conserved between closely related yeasts and the polarity of the subterminal repeats differing between TIR-L and TIR-R (Supplementary Fig. S4). This sequence was present in the host DNA downstream from TIR-R, but it was not present in the host DNA upstream from TIR-L. We speculate that this sequence might stimulate Kat1 activity. In support of this idea, a 40-bp duplex of TIR-R was a good substrate for Kat1 but a 40-bp duplex of TIR-L was not. Similarly, in the mariner transposon, nucleotides flanking the TIRs are contacted by the transposase and facilitate the cleavage reaction^49^. Hermes and other TEs have multiple subterminal repeats within their transposon ends^17^,^22^,^23^ indicating that such sequences are general facilitators of TE/transposase interactions.

The formation of hairpins involves two distinct steps. Kat1 first nicks one strand exposing a 3′-OH that in the second step attacks the phosphate backbone of the opposite strand, generating the hairpin. The second transesterification step requires a distortion of the DNA backbone of the opposite strand. In *E. coli* Tn5, it was shown that a Trp-residue performs “base flipping” in which a particular base becomes extrahelical allowing distortion of the backbone^39^. W319 in Hermes has been implicated, and W298 in Tn5 and W265 in Tn10 have been shown to be involved in a stacking interaction to the flipped-out base^18^,^32^,^50^,^51^. However, in Rag1 aromatic residues, although being important, does not appear to stack against the flipped-out base, indicating that the Tn5 model may not apply to eukaryotic transposes. Instead it was suggested that the flipped-out base was accommodated within a non-specific cleft of the recombinase, which was consistent with that the flipped-out base could be either a purine or pyrimidine^41^. W576 in Kat1 corresponds to W319 in Hermes. The Kat1W576A mutation results in more nicks, consistent with a role in hairpin formation. However, W576 is not essential for hairpinning as hairpins still are formed *in vitro* and Kat1W576A supports mating-type switching *in vivo*. In contrast, the mutation in the CXXH motif, present in the active site of Hermes, generated nicks in the template, but did not generate hairpins at all. None of the Trp to Ala substitutions generated displayed this phenotype demonstrating that the CXXH motif in Kat1 is more important for transesterification than W576 (Fig. 6b,c).

Most phenotypes observed in the different assays used in this study agreed well with each other, *i.e.* a mutant defective for DNA cleavage was also defective for strand-transfer and mating-type switching. However, there were some exceptions. The W576A mutant was defective for strand-transfer, but otherwise active. It is possible that this observed defect is due to that the strand-transfer assay was less sensitive than the other assays. Other examples are the W312A and F624A mutants that were inactive in all assays except when overexpressed *in vivo* (Fig. 6e). This indicates that galactose-mediated overexpression is a very sensitive assay, capable of detecting the activity of enzymes with a severely compromised function.

Our findings here establish that Kat1 shares many similarities and a related mechanism of action with Hermes and Rag1. Understanding Kat1 molecularly will possibly be useful to develop it as genetic tool and for comparing it with other transposases expanding our knowledge of TEs.

## Methods

### Reagents, enzymes and chemicals

Chemicals were obtained from Sigma Chemical Co., Amresco, GE health care (USA). DNA modifying enzymes were from New England Biolabs and antibodies from Santa Cruz Biotechnology (USA). ^32^P-labeled nucleotides were from Perkin-Elmer (USA). Supplementary Table 1 lists the oligonucleotides used, with Supplementary Fig. S1 depicting *MAT***a***1-MAT***a***2* specific oligonucleotides.

### Kat1 transposase expression and purification

The opening reading frame of *KAT1+G* was PCR amplified and cloned into the *E. coli* expression vector pGAT-2 to direct expression of N-terminally GST-tagged Kat1+G. Bacterial cultures (*E. coli* DE3 cells) harboring pGAT-2-*KAT1+G*^21^ and mutant derivatives were induced by addition of 1mM IPTG (at 16–18 °C for 18 h). Full length, truncated and mutant Kat1 was purified by a batch process from one liter of culture using glutathione Sepharose 4B. Purity and identity of eluted proteins was confirmed by SDS-PAGE. For introducing Kat1 mutations, site-directed mutagenesis was performed using the Stratagene QuickChange procedure. Mutant alleles were confirmed by sequencing. The resulting plasmid genotypes are listed in Supplementary Table 2 (*E. coli* expression plasmids) and 3 (*K. lactis* expression plasmids).

### 5′ end-labeling

The 5′ end-labeling of oligonucleotides or PCR products was performed using T4 polynucleotide kinase in a buffer containing 20 mM Tris-acetate (pH 7.9), 10 mM magnesium acetate, 50 mM potassium acetate, 1 mM dithiothreitol (DTT) and [γ-^32^P] ATP at 37 °C for 1 hr. The labeled substrates were purified using a Sephadex G-50 column and stored at −20 °C. Complementary oligonucleotides were annealed in a solution containing 100 mM NaCl and 1 mM EDTA pH 8.0 at 100 °C for 10 min followed by slow cooling.

### 3′ End-labeling

The 3′ end-labeling of oligonucleotides was performed using terminal deoxynucleotidyl transferase (Thermo fisher scientific) using Cordycepin 5′-triphosphate [α-^32^P] ATP at 37 °C for 30 min. The labeled substrates were purified using a Sephadex G-50 column and complementary oligonucleotides were annealed similar to 5′ end-labeling.

### Endonuclease assay

*In vitro* cleavage reactions using PCR-amplified DNA substrates and oligonucleotide substrates were performed by incubating end labeled DNA substrates with Kat1 in a buffer containing 50 mM Tris-HCl, 0.5 mM EDTA, 1 mM DTT, 100 μg/ml bovine serum albumin (BSA) and 5 mM MgCl_2_ at 30 °C for 3 hrs. The reaction was terminated by addition of EDTA (10 mM, pH8.0) followed by phenol:chloroform extraction and ethanol precipitation in the presence of glycogen. Finally, the dried pellet was dissolved in TE buffer. The reaction products were resolved on 6–15% native/denaturing PAGE. The signals were visualized using a Fuji PhosphorImager and analyzed with the MultiGauge software. Each experiment was done a minimum of three independent times.

### Electrophoretic mobility shift assay (EMSA)

PCR amplified DNA substrates were incubated with increasing concentrations of Kat1 protein (~5, 10, 20, 40 nM) for 60 min on ice in EMSA buffer (50 mM Tris-HCL, 0.5 mM EDTA, 1 mM DTT, 100 μg/ml BSA, 7 ng/μl single stranded DNA and 5 mM MgCl_2_). Reaction products (20 μl) were loaded immediately after adding 15% (v/v) glycerol and resolved on a 4% native PAGE at 4 °C for 6 h at 150 V in 0.5× TBE buffer. Unlabeled DNA was added as competitor using 75, 150 or 300 ng/reaction. The signals were visualized using a Fuji PhosphorImager and analyzed with the MultiGauge software. Each experiment was done a minimum of three independent times.

### Strand-transfer assay

Strand-transfer reactions were performed using PCR amplified DNA substrates of 197-bp from TIR-R containing 49-bp of transposon DNA plus 148-bp flanking host DNA. The TIR-R pre-cleaved 120-bp transposon DNA substrate was generated by PCR using KKC52 and KKC56 primers (Supplementary Table 1). Labeled DNA (~6 nM) was incubated with Kat1 transposase (~200 nM) along with pUC19 as target DNA (700 ng) in a buffer containing 50 mM Tris-HCl, 0.5 mM EDTA, 1 mM DTT, 100 μg/ml BSA and 5 mM MgCl_2_ for 3 hrs at 30 °C. The reactions were terminated by addition of TE buffer, deproteinized, followed by precipitation in the presence of glycogen. Target joining products were analyzed on a 1.5% TAE (tris acetate EDTA) native agarose gel as previously described^18^. The signals were visualized using a Fuji PhosphorImager and analyzed with the MultiGauge software. Each experiment was done a minimum of three independent times.

### GEMMA analysis

Instrumentation and methodology of GEMMA have been provided previously^28^,^29^,^30^. The proteins were equilibrated into 100 mM ammonium acetate pH 7.8 using Sephadex-25 chromatography and then diluted into a final concentration of 20 mM ammonium acetate and 0.005% Tween-20 prior to analysis. The GEMMA analysis was performed at a low flow-rate (pressure 2 Psi) to minimize the effect of remnant non-volatile salts on the analysis.

### Yeast strains, growth and manipulation

The yeast strains used in this study were described before: SAY572 (*MAT***a** *nej1*Δ) SAY1597 (*MAT***a** *kat1*Δ)^21^,^52^. Yeast strains were grown at 30 °C in medium containing 1% yeast extract, 2% peptone or synthetic complete medium lacking uracil (SC-Ura) with either 2% glucose or 2% galactose as the carbon source. Cloning was performed using standard methods^53^. The p*GAL-KAT1+G* vector was described before^21^. DNA/protein preparations and transformation of yeast and *Escherichia coli* followed standard protocols^53^,^54^.

### Mating type switching assay

Detailed description of this assay have been provided previously^21^. Briefly, genomic DNA was extracted from WT and Kat1 mutants, digested with BamHI and subjected to Southern blotting. DNA-blots were probed using a *MAT*-specific ^32^P-labeled probe and visualized using a PhosphorImager (Fuji).

### Immunoblotting

Detailed descriptions of the methods and reagents used for detection of Kat1 have been provided previously^21^. Briefly, WT and Kat1 mutants including a 13X-Myc tag on the N-terminus were overexpressed in SAY1597 (*MAT***a** *kat1*Δ). Protein lysates were prepared using alkaline lysis followed by centrifugation at 13,000 × g and electrophoresed on SDS-PAGE gel and transferred to nitrocellulose membranes.

## Additional Information

**How to cite this article**: Chiruvella, K. K. *et al.* Biochemical Characterization of Kat1: a Domesticated *hAT*-Transposase that Induces DNA Hairpin Formation and *MAT*-Switching. *Sci. Rep.* **6**, 21671; doi: 10.1038/srep21671 (2016).

## Supplementary Material

Supplementary Information
