# Supplementary material for: Biochemical Characterization of Kat1: a Domesticated hAT-Transposase that Induces DNA Hairpin Formation and MAT-Switching
Source: Sci Rep. 2016 Feb 23;6:21671. doi: 10.1038/srep21671 (PMC4763223; doi:10.1038/srep21671)
Supplement: Supplementary Information [file srep21671-s1.pdf]

Biochemical Characterization of Kat1: a Domesticated *hAT*-Transposase that Induces DNA Hairpin Formation and *MAT*-Switching

Kishore K. Chiruvella, Naghmeh Rajaei, Venkateswara Rao Jonna, Anders Hofer and Stefan U. Åström

Supplementary information

Supplementary Table 1. List of oligonucleotides used for generating substrates for DNA binding, DNA cleavage and DNA strand transfer assays

**Name**		**DNA sequence (5'-3')**	**Comments**
KKC3	Top strand	TGAATATATCCATCTTAATCAGACGTATAC AGAATTCACC	Figure 1
KKC4	Bottom strand	GGTGAATTCTGTATACGTCTGATTAAGATG GATATATTCA	Figure 1
KKC5	Top strand	TGAATATATCCATCTTAATCAGACGTATAC tacgacatcg	Figure S5
KKC6	Bottom strand	cgatgtcgtaGTATACGTCTGATTAAGATGGAT ATATTCA	Figure S5
KKC7	Top strand	CATCTTAATCAGACGTATACAGAATTCAC C	Figure S5
KKC8	Bottom strand	GGTGAATTCTGTATACGTCTGATTAAGATG	Figure S5
KKC21	Top strand	TGAAGCTATCCATCTTAATCAGACGTATAC AGAATTCACC	Figure 2
KKC22	Bottom strand	GGTGAATTCTGTATACGTCTGATTAAGATG GATAGCTTCA	Figure 2
KKC23	Top strand	TGAATATCGCCATCTTAATCAGACGTATAC AGAATTCACC	Figure 2
KKC24	Bottom strand	GGTGAATTCTGTATACGTCTGATTAAGATG GCGATATTCA	Figure 2
KKC25	Top strand	TGAATATATCACTCTTAATCAGACGTATAC AGAATTCACC	Figure 2
KKC26	Bottom strand	GGTGAATTCTGTATACGTCTGATTAAGAGT GATATATTCA	Figure 2
KKC27	Top strand	TGAATATATCCATCTTCAGCAGACGTATAC AGAATTCACC	Figure 2

KKC28	Bottom strand	GGTGAATTCTGTATACGTCTGCTGAAGATGGATATATTCA	Figure 2
KKC29	Top strand	TGAATATATCCATCTTAATCATCCGTATACAGAATTCACC	Figure 2
KKC30	Bottom strand	GGTGAATTCTGTATACGGATGATTAAGATGGATATATTCA	Figure 2
KKC31	Top strand	TGAATATATCCATCTTAATCAGACTGATACAGAATTCACC	Figure 2
KKC32	Bottom strand	GGTGAATTCTGTATCAGTCTGATTAAGATGGATATATTCA	Figure 2
KKC33	Top strand	TGAATATATCCATCTTAATCAGACGTCGACAGAATTCACC	Figure 2
KKC34	Bottom strand	GGTGAATTCTGTCGACGTCTGATTAAGATGGATATATTCA	Figure 2
KKC35	Top strand	TGAATATATCCATCTTAATCAGACGTATCAAGAATTCACC	Figure 2
KKC36	Bottom strand	GGTGAATTCTTGATACGTCTGATTAAGATGGATATATTCA	Figure 2
EBO110<br>EBO213	Forward primer<br>Reverse primer	GACATGGAAAGGAAGACATGG<br>GCTGTATCTAAAACAGGGACG	PCR for EMSA and cleavage assays (Figure 3B, Figure 6B,C)
EBO211<br>EBO213	Forward primer<br>Reverse primer	CAATTCACACCATTTGATGGTG<br>GCTGTATCTAAAACAGGGACG	PCR for EMSA and strand transfer assays (Figure 3C,D; Figure 4, Supplementa1 Figure S2)
KKC52<br>KKC55	Forward primer<br>Reverse primer	TATACGTCTGATTAAGATG<br>TGATGATAGAATAAAGAGAAG	PCR (pre-cleaved DNA substrate) for strand transfer assay (Figure 4C)
EBO122	Forward primer	CAAGTCTATTCGTATTCTTATTC	PCR for cleavage assay (Supplemental

EBO111	Reverse primer	CTATGTATCAATTCACCACC	Figure S3)

Supplementary Table 2. List of plasmids used for Kat1 expression and purification in bacteria

Plasmid	Kat1 mutation	Primers used for making mutation
pNR30	pGAT2+KAT1G	Cloning of Naghmeh 84/85 in pGAT2 by SalI
pNR31	pGAT2+KAT1G(D310A)	Site direct mutagenesis in pNR30 (Naghmeh29/30)
pNR38	pGAT2+KAT1G(D377A)	Site direct mutagenesis in pNR30 (Naghmeh31/32)
pNR45	pGAT2+KAT1G(E895A)	Site direct mutagenesis in pNR30 (Naghmeh33/34)
pNR117	pGAT2+KAT1G(W328A)	Site direct mutagenesis in pNR30 (Naghmeh187/188)
pNR135	pGAT2+KAT1G (K907A/R908A/R909A/R911A)	Site direct mutagenesis in dimerization domain of pNR30 (Naghmeh183/184)
pNR140	pGAT2+KAT1G (W576A)	Site direct mutagenesis in pNR30 (Naghmeh164/165)
pNR144	pGAT2+KAT1G (W312A)	Site direct mutagenesis in pNR30 (Naghmeh185/186)
pNR204	pGAT2+KAT1G(C130A/C133A)	Site direct mutagenesis in pNR30 (Naghmeh207/208)
pNR206	pGAT2+KAT1G(F624A)	Site direct mutagenesis in pNR30 (Naghmeh211/212)
pNR207	pGAT2+KAT1G(F898A)	Site direct mutagenesis in pNR30 (Naghmeh213/214)
pNR210	pGAT2+KAT1G(S899A)	Site direct mutagenesis in pNR30 (Naghmeh219/220)
pNR213	pGAT2+KAT1G(S906A)	Site direct mutagenesis in pNR30 (Naghmeh221/222)
pNR217	pGAT2+KAT1G(S886A)	Site direct mutagenesis in pNR30 (Naghmeh223/224)
pNR221	pGAT2+KAT1G(Y578A)	Site direct mutagenesis in pNR30 (Naghmeh205/206)
pNR225	pGAT2+KAT1G(C402A/H405A)	Site direct mutagenesis in pNR30 (Naghmeh209/210)

Supplementary Table 3. List of plasmids used for Kat1 expression in yeast

Plasmid	Kat1 mutation	Primers used for making mutations
pEB155	pCXJ18- pGREG526	pGREG526 module (EBO320/321) incorporated in pCXJ18 (HindIII& XbaI) by HR
pNR6	PEB155+KAT1G	Naghmeh167/EBO313 by drag and

		drop cloning
pNR122	PEB155+KAT1G(W312A)	Site direct mutagenesis in pNR6(Naghmeh185/186)
pNR126	PEB155+KAT1G(W868A)	Site direct mutagenesis in pNR6(Naghmeh189/190)
pNR131	PEB155+KAT1G(W328A)	Site direct mutagenesis in pNR6(Naghmeh187/188)
pNR148	PEB155+KAT1G(K907A/R908A/R909A/R911A)	Site direct mutagenesis of the part of the conserved hAT domain of pNR6 (Naghmeh183/184)
pNR151	PEB155+KAT1G (W576A)	Site direct mutagenesis in pNR6(Naghmeh164/165)
pNR208	PEB155+KAT1G(F898A)	Site direct mutagenesis in pNR6(Naghmeh213/214)
pNR228	pEB155+KAT1G(S899A)	Site direct mutagenesis in pNR6 (Naghmeh219/220)
pNR231	pEB155+KAT1G(S906A)	Site direct mutagenesis in pNR6 (Naghmeh221/222)
pNR234	pEB155+KAT1G(S886A)	Site direct mutagenesis in pNR6 (Naghmeh223/224)
pNR237	pEB155+KAT1G(Y578A)	Site direct mutagenesis in pNR6 (Naghmeh205/206)
pNR245	PEB155+KAT1G(C130A/C133A)	Site direct mutagenesis in pNR6 (Naghmeh207/208)
pNR247	PEB155+KAT1G(C402A/H405A)	Site direct mutagenesis in pNR6 (Naghmeh209/210)
pNR248	PEB155+KAT1G(F624A)	Site direct mutagenesis in pNR6(Naghmeh211/212)

Supplementary Table 4. List of primer(s) sequences used for site-directed mutagenesis

Name	DNA sequence (5’-3’)
EBO313	GCGTGACATAACTAATTACATGACTCGAGGTCGACCTAGGATTCT
EBO320	GTCACGACGTTGTAAAACGACGGCCAGTGAATTCGAGCTCGGTAC

EBO321	GAGCGGATAACAATTTCACACAGGAAACAGCTATGACCATGATTA
Naghmeh84	GGATCGTCGACATGATATCATCGAGTTTACATAATTTG
Naghmeh85	GGATCGTCGACCTAGGATTCTGTTTTACGTAC
Naghmeh164	GTTAGAACAAGAGCGACATATTCTGTG
Naghmeh165	CACAGAATATGTCGCTCTTGTTCTAAC
Naghmeh167	GAATTCGATATCAAGCTTATCGATACCGTCGACAATGATATCATCGAGT TTACATAATTTG
Naghmeh183	CGATCCTTACAAGTGCGGCAGCGGGAGCGATCTCACCTACAAG
Naghmeh184	CTTGTAGGTGAGATCGCTCCCGCTGCCGCACTTGTAAGGATCG
Naghmeh185	GTTTTAGATCATGCGTCAGACACAAGG
Naghmeh186	CCTTGTGTCTGACGCATGATCTAAAAC
Naghmeh187	GTTATTGTGATTGCGGATAAATACC
Naghmeh188	GGTATTTATCCGCAATCACAATAAC
Naghmeh189	CATCACAAGATTGCGGCTGATATC
Naghmeh190	GATATCAGCCGCAATCTTGTGATG
Naghmeh205	GTTAGAACAAGATGGACAGCTTCTGTGTTATGTTTTGAAAG
Naghmeh206	CTTTCAAAACATAACACAGAAGCTGTCCATCTTGTTCTAAC
Naghmeh207	GATAATAATCTGTACAGAGCTATGCTAGCCTCCATGGTGTTAAAAG
Naghmeh208	CTTTTAACACCATGGAGGCTAGCATAGCTCTGTACAGATTATTATC
Naghmeh209	CTTCATTTAAGCGCTGTTAATGCTTCATTGAATGTC
Naghmeh210	GACATTCAATGAAGCATTAACAGCGCTTAAATGAAG
Naghmeh211	CTTTTGAAACCATGCCAGACGATAACAG
Naghmeh212	CTGTTATCGTCTGGCATGGTTTCAAAAG
Naghmeh213	CATATCGAACATATCGCCAGCATATCGTCGATC
Naghmeh214	GATCGACGATATGCTGGCGATATGTTCGATATG
Naghmeh219	CATATCGAACATATCTTCGCCATATCGTCGATCCTTAC
Naghmeh220	GTAAGGATCGACGATATGGCGAAGATATGTTCGATATG
Naghmeh221	CGTCGATCCTTACAGCTAAAAGGAGAGGAAG

Naghmeh222	CTTCCTCTCCTTTTAGCTGTAAGGATCGACG
Naghmeh223	CAATCACCTAATGGACGCCATTGCTGTTTCATGAC
Naghmeh224	GTCATGAAACAGCAATGGCGTCCATTAGGTGATTG

GGTTGGTCGATGGATTTTCGTGGTCCGTTTTTTAAGTGGAAGTGTAGGGTGT

EBO 122

CAGTTTGACTTTACTATTATTCTCTTAATTATATACAAGTCTATTCGTATTCT

TATTCAAATTACCTCAAAAAAAAATGACAATATATCGAAGGACATATATAAAA

EBO 110

GATAAGACATGGAAAGGAAGACATGGTTGGTCTGATTTCATTAACGTTTCAG

GTATACATCAAAATTCAGTGAATTTAATTCTGTCGTTTGAAACCAGTTTAGGT

GAAATTGGATTAGGTGGTGAATTGATACATAGTAAATAATTACTGGTCCATAA

Ebo 111

TTTTTGTGAATATTTATGCTCTTTATACTATGTAGTTTTTATCCTGAATATTA

ACGATTGCTCACCTAAATTTCTCGTTTAAAATCCCTATAATGTGAATGGTTTT

AGATGATTCATGGTGTTTTTATTAGTGACCGAAATGTTTAGTTTTGGATACTT

KKC55

GATGATAGAATAAAGAGAAGTGTTATGTAAAAAGTGTTAATTCATGTTAAATT

EBO 211

CACTACCTTCTTGAGATTCAATTCACACCATTTGATGGTGAATATATCCATCT

TAATCAGAC**GTATAC**AGAATTCACCTTCAGATGTGTAAGAGTTTTATGTATTC

KKC52

CAATATGTCATTTGTCAATTTGTCTGCATTTCAAACAAAGGAAAAATAAACTT

AAGGACATATATCTGAAAATATACCACATAGATACGTCCCTGTTTTAGATACA

EBO 213

GCATTTTCTTGGTACAAAATCCAAACATGGATATTCCAATAAATACCCTCATG

TTGTCAACTATGCTCAAGGAATAGCCTCACAAACCTAAATCCTCAGCATTGAC

CGGAGTC

Supplementary Figure 1. DNA sequence and oligonucleotides used in the *MAT***a***1-MAT***a***2* intergenic region. Arrows indicate the positions of oligonucleotide primers used for generating PCR amplified DNA substrates for DNA cleavage, DNA binding and DNA strand-transfer analyses. GTATAC at the beginning of the TIRs is shaded. The full set of oligonucleotides used are shown in Table S1.

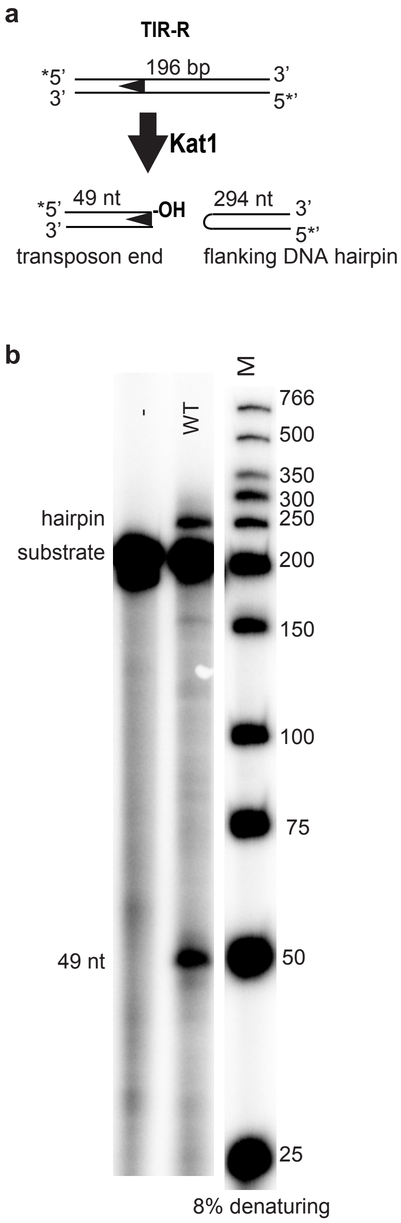

Supplementary Figure 2. Kat1 generates hairpin-capped DSBs. (A) *In vitro* cleavage assay using a 196-bp DNA substrate from TIR-R (generated using EBO211 and EBO 213), schematically shown. TIR indicated as black arrow head. The DNA substrate was mixed with GST-Kat1 or without protein (-) followed by separation on 8% denaturing PAGE. The substrate, hairpin and linear products are indicated. Both strands of the substrate were ^{32}P-labeled in their 5′-ends. (B) Kat1 cleavage resulted in flanking DNA hairpin formation by intramolecular transesterification, expecting to generate a hairpin (~294-nts) and a ~49-nt linear product. Note that these conditions do not allow nucleotide resolution of the products. M indicates a 25-bp DNA ladder.

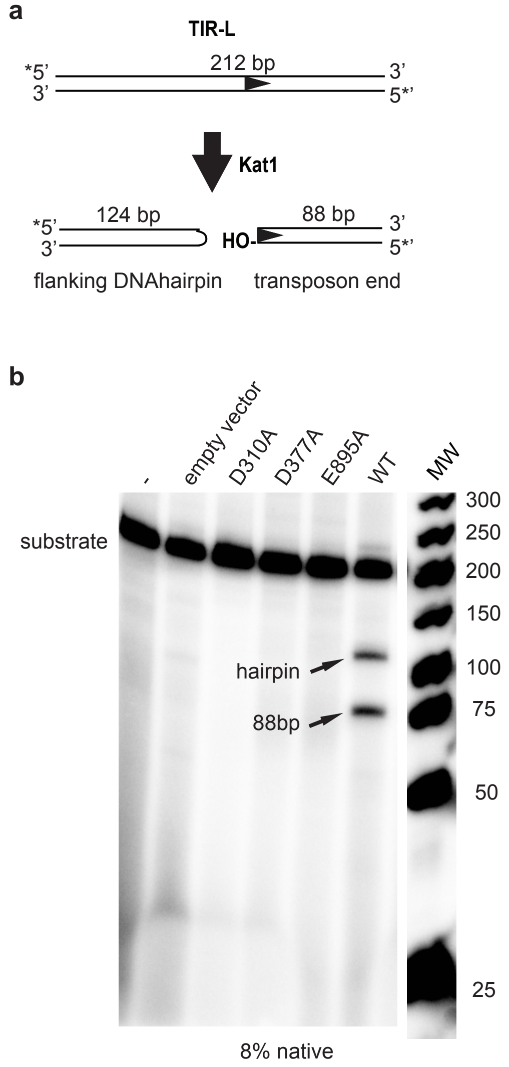

Supplementary Figure 3. Kat1 cleaves TIR-L *in vitro*. (A) *In vitro* cleavage assay using a 212-bp DNA substrate from TIR-L (generated using EBO111 and EBO122) schematically shown. TIR indicated as black arrow head. The expected lengths of the products are shown. (B) The DNA substrate (~4 nM) was mixed with GST-Kat1, the indicated DDE-mutants or without protein (-) followed by separation on 8% PAGE using native conditions. Kat1 cleavage resulted in flanking DNA hairpin formation by intramolecular transesterification, expecting to generate a hairpin of ~124-bp and a ~88-bp linear product. M indicates a 25-bp DNA ladder.

```
lactis          AAGACATGG----AAAGGAAGACATGGTTGGTCTGATTTCATTAACGTTTCAGGTATACA 218
dobzhanskii     AAGACATGGTGAAAAAAAAAGACATGTTAGCTTTGATCTCATTCTGGTTTCAAGTATACA 239
marxianus       CAGAGAGAG----CTAAGACATAATGTTT--TAAGATTTCATTTTAGTTTCAGGTATACA 217
                .*** * .*    .:*..*..:.*** *:  * :*** ***** : ******.*******

lactis          TCAAAATTCAGTGAATTTAATTCTGTCGTTTGAAACCAGTTTAGGTGAAATTGGATTAGG 278
dobzhanskii     TCATAATCGAGTGAATTTGAATCTATCG-CTCAGTTGAATTTGGGTGAACTTGAAGTACT 298
marxianus       TCAAATTTTAATGAATTCAATTGTCCTTCTTGACTAGTTTCTTGATGGATTCTGAGTACT 277
                ***:*:*  *.****** .*:* *      * * :  : * * *.**.* *  .* **

lactis          TGGTGAATT-GATACATAGTAAATAATTACTGGTCCATAATTTTTGTGAATATTTATGCT 337
dobzhanskii     TGGTGAATTTAATGCATAGTAATTAAATAAT---CTATACCGTCGTTGGGTAAAAATACA 355
marxianus       TGGTGAATTAAAGTAATGGTTTACTATGTAT----TTTGAAGTTTTAG---CTATCCGAC 330
                ********* .*  .**.**::: :*: :.*     :*..  *   :*   .:::. ..

lactis          CTTTATACTATGTAGTTTTTATCCTGAATATTA--ACGATTG---CTCACCTAAATTTCT 392
dobzhanskii     GAGAGTACTTTTTGTTTGAAATACTCAATTAAAG-TCAGTAGTTTTTAGCTTATTTTGT 414
marxianus       GACAGTTAACTTTATTAAATAAAATAAATCTTACTACTATTG-TTAATTTATAATTCCGT 389
                 : :.*:.: * *. *: ::*:..* *** ::*  :* .*:*    : : .*:*:*   *

lactis          CG--TTTAAAATCCCTATAATG----TGAATGGTTTTAGATGATT-CATGGTGTTTT-TA 444
dobzhanskii     CG--TTCGATTTCACACTGTAG----CAGTCCATTTCTCCTCAGCGTATCGTTTTTT-TG 467
marxianus       CTGAATAAATTTCTAATTTTTGGTATATATCTGTCTTTGGTAGTCCTAAATAACATAGTT 449
                *   :* .*::** .: * ::*      .:  .* * :  * .    *:  :  :*: *

lactis          TTAGTGACCGAAATGTTTAGT-TTTGGATACTTGATGATAGAATAAAGAG--AAGTGTTA 501
dobzhanskii     CTGGTATCAGAAAGATGAAAT-ATTGCTGAAGTTATCGTGACAGTAAGTC--AACAGCTC 524
marxianus       TTGGAAGCTGTTTGATATCTTGTAAACTGATTTAAATATACCAAAAATTCATAAGAGAGC 509
                 *.*:. * *::: .* :. * :::. : *  * *: .*.  .*:** :   ** :*  .

lactis          TGTAAAAAGTGTTAATTCATGTTAAATTCACTACCTTCTTGAGATTCAATTCACACCATT 561
dobzhanskii     ATTCAAAAGTGTTAATTCTGGTAGAATTCACCAACTTCTTGAGAGTCAATTCACTACAAT 584
marxianus       TTAATAAATGGTTAATTCATCAGAAATTCACCAGTTCTTCATAAATGAAATCACCAACTC 569
                : :.:***  ********:  : .******* *  *  * .:.* * **:**** ...:

lactis          TGATGGTGAATATATCCATCTTAATCAGACGTATACAG--AATTCACCTTCAGATGTGTA 619
dobzhanskii     TGAAGGTGAATACATCCACCTAAATCAGATGTATACAGGAAATACATGTTGTGATG-CAG 643
marxianus       TTCGGGTGAATTTATTCACCATATTTTGCTGTATACTA----AACTAATGAAAACCCTTC 625
                * . *******: ** ** *::*:* :*. ******:.    ::*:  *  :.*    :
```

TGAATT - subterminal repeat, AATTCA-complement of subterminal repeat,

GTATAC - Beginning of TIRs

Supplementary Figure 4. Multiple sequence alignment of the *MAT***a***1*-**a***2* intergenic region in *K. lactis*, *K. dobzhanskii* and *K. marxianus*. Identical bases in all three species are indicated by asterisks. GTATAC in blue represents the beginning of TIRs. 5'-TGAATT (yellow) and its reverse complement (red) are subterminal repeats.

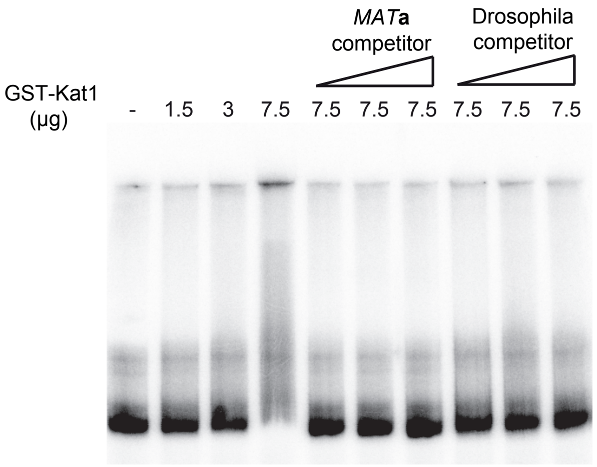

Supplementary Figure 5. Kat1-DNA binding is not sequence specific. EMSA was performed with *MAT***a***1-MAT***a***2* intergenic region (579 bp) covering TIR-L and TIR-R, with increasing amounts of Kat1 for 1h at 4^0C. Identical amounts (75, 150 and 300ng/reaction) of two different competitor duplexes, same as the probe and Drosophila DNA (570 bp) were used to compete the bandshift.

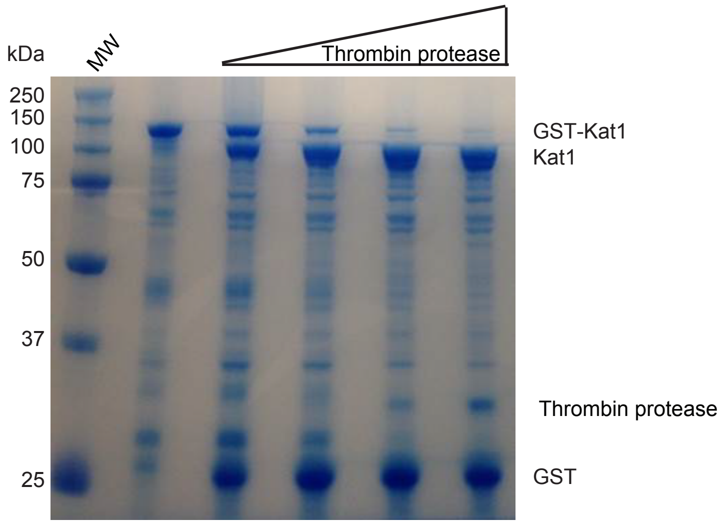

Supplementary Figure 6. Thrombin protease cleavage of GST-Kat1. Coomassie-stained SDS-PAGE showing cleavage of the GST tag from the GST-Kat1 fusion protein. Approximately 10 µg of Kat1 was incubated with increasing concentrations (0.5, 1, 2.5, 5, 10 units) of thrombin protease for 22 hours at 4°C. The molecular weight (MW) is shown on the left. GST-Kat1, Kat1, GST and thrombin protease are indicated on the right.

```
                                           130 133
Kat1      SRPYKGTVQAVWSYFK----------ENADNNLYR-CMLCSMVLKV--EKH-QNGNTVRI 150
Hermes    -RHK----GTSFIWNVLADIQKE---DDTLVEGWVFCRKCE-----KVLK--YTTRQTSN 67
Tfo1      KRTLNSKLQPSAIYKHGA-----QLTTDGDNKYWL-CKYCHIRGHHHTALFS--SESTTS 144
Drifter   --------------AHGYNVQAKTATTERGPSSWL-CCHCTQQKAHKPKA--YVASNTRN 43
Restless  --TKGA--KVAWWWVKGFRMRLK---SNDKKLRWV-CRLCVRRKCRTVSHFSYESNGSAN 132
                                     :     :  *  *

            310 312              328
Kat1      LVLDHWSDTR-LRSFIGVVIVIWDKYQKKQRSFVIGMPETVN--HSSAAIKDQLEQVIQH 363
Hermes    ATIDLWTDNYIKRNFLGVTLHYHE--NNELRDLILGLKSLDFERSTAENIYKKLKAIFLQ 234
Tfo1      ISVDAWTSEE-GTNYLAVVAHFLD-ESHKLQTALLDLPPLKG-PHSGENLAKALSKVIDF 325
Drifter   IAFDGWTSRN-RHSFFSINAFFLDDETFQPRKILLGLPNVAM-AHTGENICAAVTEVLEE 235
Restless  IAFDGWTSRN-QLSLLGVNCFFVD-QLWRHRRLLLALPAVSG-RHTGDNLANEVADVLAE 316
            .* *:.     . :.:     :    . :  :: :        :.  :   :  ::

                       377                         402 405
Kat1      YPGLNKM-IISSAADNASSVKNACLGLTSQCPD---RLLHLSCVNHSLNVVNSKLVTEPS 419
Hermes    FNVEDLSSIKFV-TDRGANVV-----------KSLANNIRINCSSHLLSNVLENSFE--- 279
Tfo1      YDIS--TVIGFFMMDNAGNNDTCIQELAKQYPAIKP-QSRLRCVGHMLNLIVKALLFGQG 382
Drifter   FELVQHNKLGYFVLDNASNNDKAVEELGRKFEWHEPAARRIRCFGHVLHLVATAMLFVHD 295
Restless  WDLG-SDRLGYMVLDNASNNDTAMVALGKEFGF-DPDERRLRCLGHVINLAVKQLIFGEA 374
          :       :     *....                    :: *  * :       .

                                             576
Kat1      LEIECRRPVNRKAIKKYCTDKRLGDIDGLIPYVRTRWTYSVLCFERATLLAPCLLQLIKE 599
Hermes    ----------LQH----------RLRSSLKSECPTRWNSTYTMLRSILDNWESVIQILSE 342
Tfo1      GSIEC-------------------LYTRVLVDGGIRWNSAYAMIERALKLRHAIDLFFLN 479
Drifter   -------PD-KNY----------PGTLDVVLDNCTRWLSQYYMIERAIKLRRYLEELVDI 385
Restless  GVLERIDPETGKK----------RVPLRPIADNETRWNSRHRMMVRALLLRRYLNRIVEK 489
                                             **      :         :  :.

                                                        624
Kat1      G-------------------------SLSSLLDEFDFECIRVYIILLKPFQTITAFFNSP 634
Hermes    AGETQRI-------------------------VHINKSIIQTMVNILDGFERIFKELQTC 377
Tfo1      YNHIGK-----------------EYDISQDMLTPQDWVDLEHFLGILKPFKDLTKRMEGR 522
Drifter   TIQTNRKLARSRSKVEKSRSSLPSCLEEDNLLTDTDWEALNWFSNILAMFNFCLLRLEGD 445
Restless  AERAW---ERSK-----RKSVKPSI--LDDKLSEEDWDVVEVFIQVLRPFDEISVRLQGN 539
                                             :   :.    :*  *.     ::

                         868                886      895 898899
Kat1      YEYLLLTMRDV-------HHKIWA-DIKNCPILSLFNHLMDSIAVSSTHIEHIFSISSIL 904
Hermes    FEFYRKEI--VILSEDFKVMEWWNLNSKKYPKLSKLALSLLSIPASSAASERTFSLAGNI 581
Tfo1      LDEFMARA-NRADVEVEDPLEWWVCHASDYPILSKMAFDLFSCPAMSAECERVFSQTKKV 748
Drifter   FERWQSTKQD-TFSKHDNPLEYWSAKRFEYPRVAKMAIDVLSVPAMAAECERAFSSASSM 663
Restless  YERYIQTFTHADDKYQFRPLSWWQEHEMEYPNLCRMATDLLSIPTMSAETERSFSSAGKM 770
           :                  . *  .  .  * :.  :   :  *  . ::  *: ** :  :

          906 907-909,911
Kat1      TSKRRGRISPTSLEKRMKAKIA---YMALGNYHKFDLKSTSLDQILFVRKTES        954
Hermes    ITEKRNRIGQQTVDSLLFLNSFYKNFCKLDI----------------------        612
Tfo1      ITDERNRLKSDTVAALECQKHLLRTGMLP------------------------        777
Drifter   VSPQRTRLDASTIAVTQTVRSWLKAGLLEGYDGLLKETSGEVAGVVT------        710
Restless  VSPLRTRLDRHTIGMAQGMRSWSREGIVLPSW---------------------        802
           :  * *:   ::      .
```

Supplementary Figure 7. Conserved residues in Kat1. Multiple sequence alignment (ClustalW) of Kat1, Tfo1, Drifter, Restless and Hermes (41-44). Because of low sequence similarity, the alignment of the Kat1 N-terminus (1-104) and part of the insertion domain (634-852) was omitted. Conserved amino acid residues are marked by asterisks (*). Amino acids changed by site-directed mutagenesis in Kat1 are labeled in red.

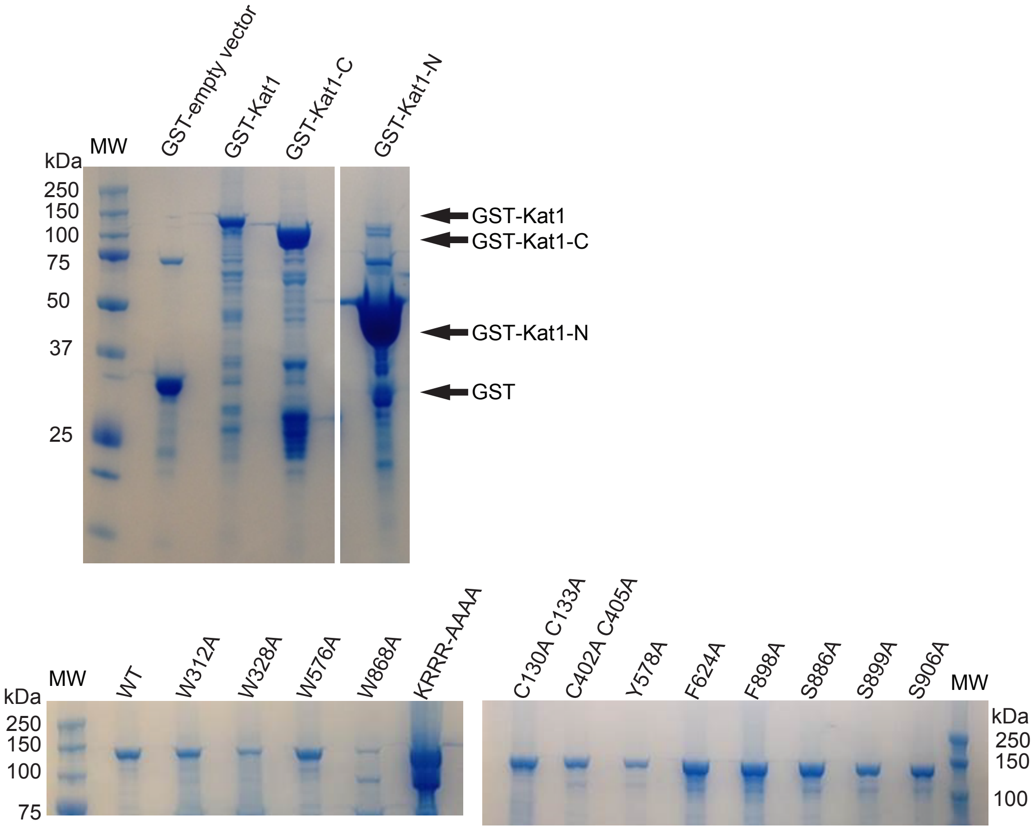

Supplementary Figure 8. Kat1 mutant proteins are stable *in vitro*. Coomassie-stained SDS-PAGE showing recombinant GST (26 kDa), GST-Kat1 (136 kDa), GST-Kat1-C (amino acids 201-958, 113 kDa), GST-Kat1-N (amino acids 1-200, 49 kDa), and GST-Kat1 proteins with indicated amino acid substitutions (bottom panel). The molecular weight marker (MW) is indicated. Table S2 describes the plasmids used for expressing the proteins.

a

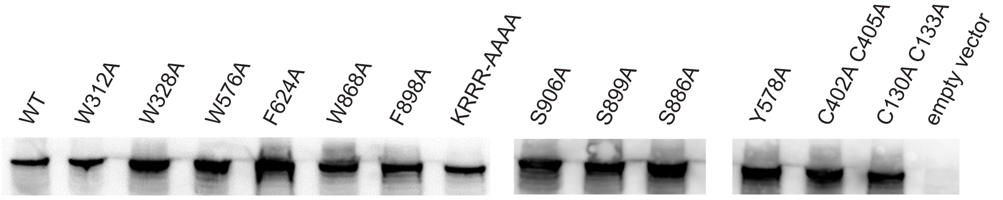

b

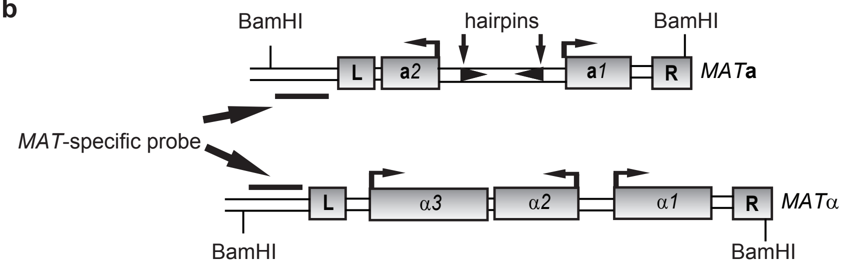

Supplementary Figure 9. (A) Kat1 mutant proteins are stable *in vivo*. Protein-blot analysis using whole cell extracts from a *MAT***a** *kat1*Δ strain (SAY1597) containing the empty vector or plasmids expressing Kat1, and Kat1 mutants. An anti-Myc antibody (9E11) was used to detect expression of Myc-tagged Kat1. The equivalent of 0.2A_{600} units of cells were loaded in each lane. Table S3 describes the plasmids used for expressing the proteins. (B) Schematic drawing of the *MAT***a** and *MAT*α loci with the BamHI-sites indicated and the sites of Kat1 cleavage shown (arrows). Boxes denote genes and the repetitive elements Left (L) and Right (R). The *MAT*-specific probe is also indicated.
